# Self-generated oxygen gradients control collective aggregation of photosynthetic microbes

**DOI:** 10.1098/rsif.2021.0553

**Published:** 2021-12-01

**Authors:** Alexandros A. Fragkopoulos, Jérémy Vachier, Johannes Frey, Flora-Maud Le Menn, Marco G. Mazza, Michael Wilczek, David Zwicker, Oliver Bäumchen

**Affiliations:** ^1^ Max Planck Institute for Dynamics and Self-Organization (MPIDS), Am Faßberg 17, 37077 Göttingen, Germany; ^2^ Nordita, KTH Royal Institute of Technology and Stockholm University, Hannes Alfvéns väg 12, 106 91 Stockholm, Sweden; ^3^ Interdisciplinary Centre for Mathematical Modelling and Department of Mathematical Sciences, Loughborough University, Loughborough, Leicestershire LE11 3TU, UK; ^4^ Experimental Physics V, University of Bayreuth, Universitätsstr. 30, 95447 Bayreuth, Germany

**Keywords:** microbial motility, photosynthesis, oxygen respiration, collective effects, living active matter

## Abstract

For billions of years, photosynthetic microbes have evolved under the variable exposure to sunlight in diverse ecosystems and microhabitats all over our planet. Their abilities to dynamically respond to alterations of the luminous intensity, including phototaxis, surface association and diurnal cell cycles, are pivotal for their survival. If these strategies fail in the absence of light, the microbes can still sustain essential metabolic functionalities and motility by switching their energy production from photosynthesis to oxygen respiration. For suspensions of motile *C. reinhardtii* cells above a critical density, we demonstrate that this switch reversibly controls collective microbial aggregation. Aerobic respiration dominates over photosynthesis in conditions of low light, which causes the microbial motility to sensitively depend on the local availability of oxygen. For dense microbial populations in self-generated oxygen gradients, microfluidic experiments and continuum theory based on a reaction–diffusion mechanism show that oxygen-regulated motility enables the collective emergence of highly localized regions of high and low cell densities.

## 1. Introduction

Photosynthesis is a fundamental process of life that converts light into chemical energy [1]. As a metabolic process, it is ubiquitous in highly diverse groups of species, ranging from higher level plants to prokaryotic cyanobacteria [2] and eukaryotic microalgae [3]. These unicellular photosynthetic microbes inhabit almost any ecosystem on our planet and are dispersed to heterogeneous microhabitats. Their regulation, acclimation and adaptation to light are key to the survival and propagation in their environment [4]. Light is perceived by photoreceptors [5], which control a multitude of essential biological functionalities including circadian rhythm [6–8], sexual reproduction [9], directed motion along a light gradient (phototaxis) [10–13], and adhesion to surfaces [14].

The photosynthetic machinery itself is regulated by the light harvesting complex of chlorophyll a/b, representing another pathway to respond to light cues [15]. In unfavourable light conditions, photosynthetic microbes can still survive and produce energy through aerobic respiration, allowing them to produce ATP by the consumption of oxygen [16]. This consumption however can result in self-generated dark anoxia [17,18], where the cell is deprived of both light and oxygen. Light directly controls the activities of both metabolic pathways, photosynthesis and aerobic respiration, and thus has profound implications on the life of microbial populations. We show that, by inhibiting photosynthetic activity, a confined suspension of *Chlamydomonas reinhardtii* cells can form large-scale aggregations. Collective aggregation is controlled via the microbial motility, which sensitively depends on the availability of light and oxygen. The appearance as well as the dynamics of aggregation are governed by the light intensity and wavelength as control parameters, providing a direct link between the activity of the photosynthetic machinery, microbial motility and large-scale self-organization.

## 2. Aggregation of photosynthetic microbes at low light intensity

**Figure 1.**
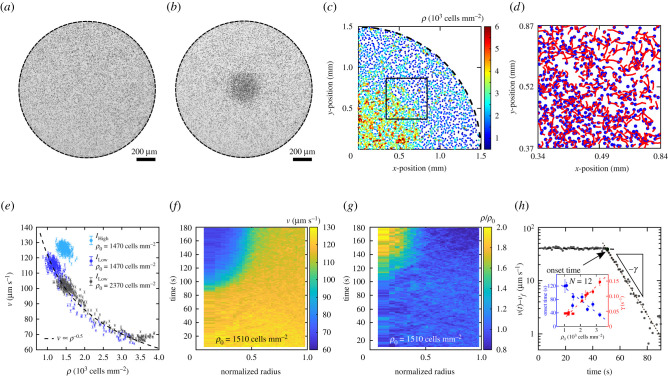
Light-switchable microbial aggregation of confined *C. reinhardtii* suspensions. Planktonic *C. reinhardtii* cells are confined in a transparent cylindrical compartment (radius *R* = 1.5 mm, height *h* = 21 μm) and illuminated with red light (*λ* = (671 ± 5) nm) of controlled light intensities. Top and bottom walls are impermeable to air, whereas the sidewalls allow for air exchange. (*a*,*b*) Top view of a suspension of *C. reinhardtii* cells of global cell density *ρ*_0_ = 1300 cells mm^−2^. The microbes are homogeneously distributed (*a*) for a light intensity *I* = 20 μmol m^−2^ s^−1^ (referred to as high), and separated into a dense phase in the centre and a dilute phase otherwise (*b*) for *I* = 0.37 μmol m^−2^ s^−1^ (referred to as low). (*c*,*d*) Cell tracking analysis for an experiment at *I* = 0.37 μmol m^−2^ s^−1^ and *ρ*_0_ = 2370 cells mm^−2^. (*c*) Snapshot with cell positions colour-coded according their local cell density *ρ* measured by Voronoi tessellation. See also electronic supplementary material, movies 1,2. (*d*) Snapshot with cell positions (blue dots) and their trajectories (red lines) over 0.5 s, corresponding to the square box shown in (*c*). See also electronic supplementary material, movies 3,4. (*e*) Velocity-density coupling *v*(*ρ*) for different light intensities *I* and global cell densities *ρ*_0_. The dashed line corresponds to *v* ∝ *ρ*^−0.5^. Error bars show the standard error of the local *v*. (*f*,*g*) Emergence of microbial aggregation as evidenced by the spatio-temporal evolution of *v* and *ρ*, after switching to low light intensity at time *t* = 0 s. (*h*) Semi-log representation of the cell velocity *v*(*t*) at the centre of the compartment, where *v*_f_ denotes the steady state velocity within the dense phase. Lines indicate best linear fits to the experimental data before (dashed) and after (dotted) the onset of aggregation; their intersect determines the onset time *t*_on_. The inset displays onset time *t*_on_ and velocity decay rate *γ* as a function of *ρ*_0_; dashed lines represent best linear fits to the data. Error bars show the 95% confidential interval of the best linear fits. *N* indicates the number of independent experiments.

A suspension of motile *C. reinhardtii* cells is confined in a transparent circular compartment of radius *R* = (1.5 ± 0.05) mm and height *h* = (21 ± 1) μm, as displayed in figure 1*a*,*b* and electronic supplementary material, figure S1a, such that top and bottom walls are impermeable to air and sidewalls allow for air exchange. This quasi-2D confinement enables identifying and tracking a large number of microbes in their planktonic, i.e. free swimming, state under controlled light and air exchange conditions. Experiments are performed using bright-field microscopy and red light (wavelength *λ* = (671 ± 6) nm) in order to safely inhibit phototactic effects [19] and surface attachment of the cells [14]. When a dense suspension of *C. reinhardtii* cells is exposed to a light intensity *I* = 20 μmol m^−2^ s^−1^, in the following referred to as ‘high’ light intensity, we find that the cells are homogeneously distributed in the compartment, see figure 1*a*. Remarkably, when the light intensity is decreased to *I* = 0.37 μmol m^−2^ s^−1^, referred to as ‘low’ light intensity, the same community of motile cells exhibits a spatially inhomogeneous distribution featuring a substantially enhanced cell density in the centre of the compartment, see figure 1*b*. This effect is completely reversible when the light condition is reverted (see electronic supplementary material, figure S2).

Besides spatially localized density variations (figure 1*c*; electronic supplementary material, movies 1,2), we also find that microbial motility exhibits spatial variations, with cells at high local cell densities moving slower than their counterparts at lower cell densities (figure 1*d*; electronic supplementary material, movies 3,4), which points to a collective effect mediated by reduced swimming velocity. This speed reduction is unrelated to photokinesis [20], where the velocity depends on the light intensity, since all cells are exposed to the same light conditions and yet exhibit different speeds. However, a generic coupling of swimming motility and local density becomes evident by linking the local cell density, *ρ*, and the local root-mean-square (rms) speed of the cells, *v*. At high light intensity, the *v* versus *ρ* data are highly localized, exemplifying the fact that the system is homogeneous (figure 1*e*). By contrast, we observe a bimodal distribution of velocities and densities for low light intensity, representing the dense phase in the centre and the dilute phase elsewhere in the compartment (see electronic supplementary material, figure S3). Notably, the transition between both phases follows a power law with *v* ∝ *ρ*^−0.5^, independent of the global cell density of the suspension. The direct comparison of the different steady states between high and low light intensity for the same cell density demonstrates that the emergence of the spatial pattern is directly coupled to modulation of microbial motility at different light intensities.

The local density *ρ* and the swimming velocity *v* are now monitored as a function of both time and distance from the centre of the compartment, see figure 1*f*,*g*. The swimming velocity *v* is initially independent of the position within the compartment, as expected for the homogeneous state. Initially, we find that *v* linearly decreases in the entire compartment at a comparably small constant rate of 0.08 ± 0.03 μm s^−2^ (dashed line in figure 1*h*). At a characteristic onset time *t*_on_ in the range of tens of seconds, the swimming velocity in the centre of the compartment strongly decreases, see figure 1*f*. This region of reduced microbial motility continuously grows in size until after a few minutes it reaches a steady state, characterized by a well-defined final size (see electronic supplementary material, figure S4). In accordance with the *v*(*ρ*) coupling for the steady states, the local cell density increases in the centre of the compartment, with a time lag of about 4–7 s to *t*_on_, see figure 1*g*. This sequence of events shows that the change in light intensity has an effect on the microbial motility, which subsequently, via the generic *v*(*ρ*) coupling, manifests in changes of the cell density. The kinetics of the aggregation process are characterized by the local velocity in the centre of the compartment decreasing exponentially with time, i.e. *v* − *v*_*f*_ ∝ e^−*γt*^, where *γ* is the velocity decay rate and *v*_*f*_ the steady-state velocity within the dense phase, see figure 1*h*. This aggregation phenomenon is of a truly collective nature, as onset time *t*_on_ and decay rate *γ* are directly linked to the global cell density, see inset of figure 1*h*. These timescales were measured repeatedly for the same experiment without noticeable differences, indicating that the phenomenon does not exhibit strong acclimation, as it has been observed before in the case of phototaxis [21].

## 3. Inhibition of photosynthesis regulates microbial motility and aggregation

The motility of each individual *C. reinhardtii* cell is driven by its continuous and coordinated flagella beating, which is fuelled by the conversion of ATP into mechanical stresses along the flagellar axoneme via molecular motors [22]. *C. reinhardtii* is a mixotrophic microorganism, and as such it is able to produce ATP either through photosynthesis in their chloroplast or through aerobic respiratory system via the consumption of oxygen [16,23]. Chlorophyll a and b are responsible for the light regulation of the photosynthetic activity [15,24], whereas aerobic respiration depends on the availability of oxygen in the liquid medium. In unfavourable light conditions, the photosynthetic activity of the cells is inhibited, eventually forcing their metabolism to switch from using the photosynthetic machinery to oxygen respiration [25].

**Figure 2.**
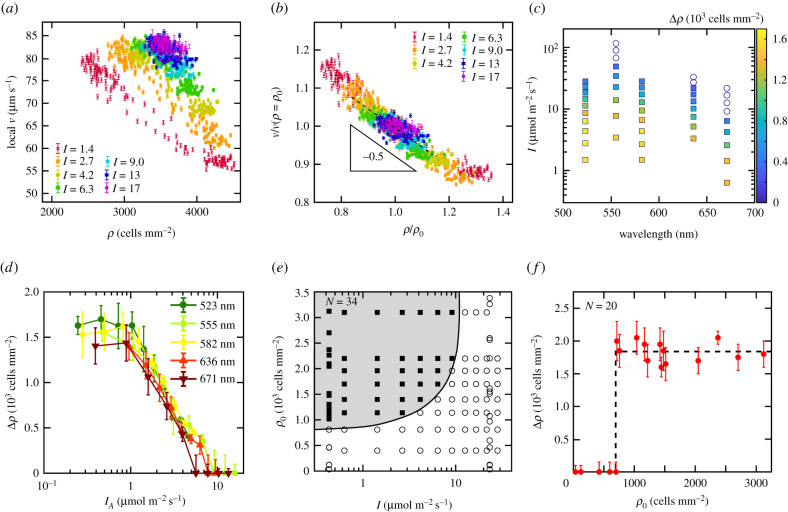
Light intensity and wavelength control the emergence of aggregation in *C. reinhardtii*. (*a*) Local cell velocity *v* versus local cell density *ρ* for different red light intensities (*λ* = 671 nm). (*b*) Scaled *ρ* and *v* of the experimental data shown in (*a*) using the global cell density, *ρ*_0_, and the velocity at the global dell density, *v*(*ρ* = *ρ*_0_), respectively. The intensities in the legends are given in units of μmol m^−2^ s^−1^. Error bars show the standard error of the local *v*. (*c*) Colour-coded representation of the order parameter, Δ*ρ*, as a function of light intensity and wavelength (open circles: Δ*ρ* = 0, filled squares: Δ*ρ* ≠ 0). (*d*) Order parameter Δ*ρ* as a function of the chlorophyll-absorbed light intensity, *I*_A_, for different light wavelengths. All results shown in (*a*–*d*) were obtained from the same *C. reinhardtii* population with *ρ*_0_ = 3400 cells mm^−2^, whereby the light conditions were changed in random order. (*e*) Phase diagram *ρ*_0_ versus *I* characterizing the critical light intensity (*λ* = 671 nm) and critical cell density of aggregation (open circles: Δ*ρ* = 0, filled squares: Δ*ρ* ≠ 0). (*f*) Above the critical cell density, Δ*ρ* is independent of the global cell density *ρ*_0_ (shown for *I* = 0.37 μmol m^−2^ s^−1^ and *λ* = 671 nm). Δ*ρ* was calculated by fitting the local cell density distribution with a bimodal distribution, and calculating the difference of the two mean densities. Error bars show the mean standard deviation of the two fitted normal distributions. *N* indicates the number of independent experiments.

To establish a link between light-regulated metabolic energy conversion, motility and aggregation, we now systematically tune the light intensity and wavelength and evaluate distributions of local velocity *v* and cell density *ρ*, see figure 2. Below a critical light intensity, the suspension gradually transitions from a homogeneous state to an aggregated state. Upon decreasing the light intensity, the initial unimodal distribution of data points splits into a bimodal distribution, where the two clusters, corresponding to the dilute and the dense phase, progressively move apart, see figure 2*a*. In addition, the overall velocity of the cells monotonically decreases with decreasing light intensity (figure 2*a*), exemplifying that the light intensity can be used as a control parameter for the cell motility. A cumulative plot of the normalized velocity, $\tilde{v}=v/v(\rho =\rho_{0})$, versus the normalized density, $\tilde{\rho }=\rho /\rho_{0}$, collapses all light intensities onto one master curve with a slope of −0.5 ± 0.1. This implies $dv(\rho )/d\rho {|}_{\rho =\rho_{0}}=-0.5v(\rho_{0})/\rho_{0}$, which is in accordance with the power-law dependence *v* ∝ *ρ*^−0.5^ shown in figure 1*e*, demonstrating that the same power law persists for all different light intensities (figure 2*b*) and global densities (see electronic supplementary material, figure S5).

In analogy to gas/liquid phase separation, we introduce the difference between the average cell densities in the dense and dilute phase Δ*ρ* as an order parameter [26] (see electronic supplementary material, figure S3*d*). Figure 2*c* displays Δ*ρ* for different light intensities and wavelengths, providing evidence that Δ*ρ* does not only depend on the light intensity (figure 2*a*,*b*) but also on the wavelength of the illumination, see figure 2*c*; the critical light intensity for aggregation is found to be about one order of magnitude smaller for red (*λ* = 671 nm) compared to green light (*λ* = 555 nm). Plotting Δ*ρ* versus the light intensity *I*_A_ absorbed by chlorophyll for each wavelength [27] collapses all data onto a master curve, see figure 2*d*, demonstrating that the inhibition of the photosynthetic activity (see electronic supplementary material, figure S6) governs the aggregation: for decreasing *I*_A_, Δ*ρ* increases and saturates at about 1500 cells mm^−2^ s. We need to note our experiments at 671 nm show that phototaxis is not responsible for the phenomenon, allowing us to perform experiments at lower wavelengths. However, we cannot perform experiments below 510 nm due to the adhesion of cells at surfaces [14].

We explore the phase space of the phenomenon by gradually varying both the light intensity and the cell density, see figure 2*e*, and thereby identify a critical cell density, *ρ*_*c*_, at about 800 cells mm^−2^ for a light intensity *I* = 0.37 μmol m^−2^ s^−1^, which increases for increasing light intensity. For *I* = 0.37 μmol m^−2^ s^−1^, Δρ displays a discontinuity at the critical cell density, *ρ*_c_, see figure 2*f*, which is strikingly different to the continuous transition observed for experiments at constant *ρ*_0_ = 3400 cells mm^−2^, see figure 2*d*.

## 4. Statistical analysis of the microbial motility in dense suspensions

Unraveling the mechanism underlying the emergence of aggregation of motile microbes ultimately requires establishing a quantitative link between light conditions, cell density and microbial motility. The motility of *C. reinhardtii* is characterized as a run-and-tumble (RT) motion [28,29], where the cell’s motion is predominantly ballistic on short timescales and exhibits sudden reorientations at a typical time *τ*_*R*_. We quantify the cell motility in dense *C. reinhardtii* suspensions using the velocity autocorrelation function [30], *C*_*v*_(*t*) = 〈**v**_*i*_(*t* = 0) · **v**_*i*_(*t*)〉, where **v**_*i*_(*t*) is the velocity of an individual cell *i* and the average is taken over all tracked cells. *C*_*v*_ follows an exponential decay (see electronic supplementary material, figure S7*a*), and we thus express the velocity autocorrelation function as $C_{v}=v^{2}\;e^{-(t/\tau_{C})}$, where $v=\sqrt{\left\langle v^{2}\right\rangle }$ is the rms velocity, and *τ*_*C*_ denotes the velocity correlation time. For RT motion [31], *τ*_*C*_ characterizes particle reorientations and is linked to the rotational diffusion constant *D*_R_ = (2*τ*_*C*_)^−1^.

**Figure 3.**
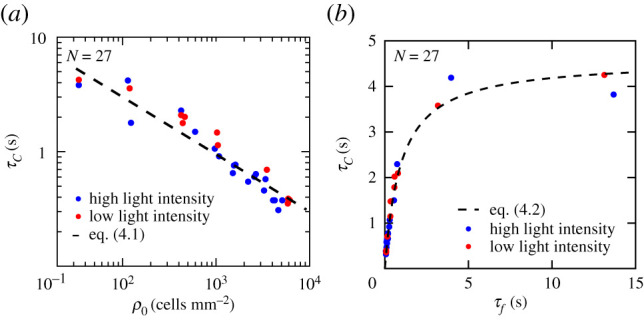
Motility analysis of dense suspensions of photosynthetic microbes. (*a*) Velocity correlation time, *τ*_*C*_, as a function of the local cell density at *I*_low_ and *I*_high_, respectively. The dashed line corresponds to *τ*_*C*_ ∝ *ρ*^−0.5^. (*b*) *τ*_*C*_ as a function of the collision time, *τ*_*f*_. The dashed line is a best fit to equation (4.2). The errors of *τ*_*C*_ and *τ*_*f*_ of a single experiment are typically within the symbol, and thus much smaller than the variability between experiments. *N* indicates the number of independent experiments.

Independent of the light intensity, *τ*_*C*_ is decreasing for increasing density, see figure 3*a*, i.e. the velocity of the cells decorrelates faster for increasing *ρ*, which results from more frequent reorientations of the motile cells due to more frequent cell–cell interactions. In the framework of ensembles of active Brownian particles in 2D, *τ*_*C*_ ∝ *τ*_*f*_ is predicted, where *τ*_*f*_ is the time between collisions. We now write the collision time as *τ*_*f*_ = *l*_*f*_/*v*, where $l_{\;f}={\left(\sqrt{8}\;d\rho \right)}^{-1}$ is the mean-free-path in 2D and *d* the cell diameter [32]. From *τ*_*C*_ ∝ *l*_*f*_/*v* and *l*_*f*_ ∝ *ρ*^−1^, together with the generic coupling *v* ∝ *ρ*^−0.5^ extracted from experiments exhibiting aggregation (see figures 1*e* and 2*b*), we expect

4.1 $$\tau_{C}\propto \rho^{-0.5},$$

which is validated by the motility analysis (figure 3*a*; dashed line). For individual *C. reinhardtii* cells, the motility is governed by their RT motion [28], featuring a characteristic tumble time *τ*_*R*_. Thus, we expect *τ*_*C*_ = *τ*_*R*_ in the density limit where the velocity decorrelates due to tumble events rather than cell–cell collisions. By adding the rotational diffusion constant for the collision-dominated and the tumble-dominated regime yields 1/*τ*_*C*_ = 1/(*cτ*_*f*_) + 1/*τ*_*R*_, where *c* is the number of collisions needed for the velocity to decorrelate. Thus, we find

4.2 $$\tau_{C}=\frac{c\tau_{f}\tau_{R}}{c\tau_{f}+\tau_{R}},$$

which quantitatively captures the experimental data using *v*, *ρ* and *d* from *N* independent experiments, see figure 3*b*. The values of *τ*_*C*_ increase linearly with *τ*_*f*_ in the collision-dominated limit, i.e. for high cell densities and small *τ*_*f*_, and saturate for large *τ*_*f*_ to a constant value representing the tumble time of a single cell [28]. A best fit of equation (4.2) to the data yields *c* = 4.8 ± 0.2 and *τ*_*R*_ = 4.2 ± 0.3 s, which is shown as the dashed line figure 3*b*. From these data, it is evident that light does not at all affect the reorientation dynamics of the planktonic microbes, but only controls their average swimming velocity.

## 5. Effect of the oxygen concentration field and continuum modelling

Having identified the interplay between inhibition of the photosynthetic activity, swimming velocity and the formation of dense and dilute phases leaves us with the question regarding the feedback mechanism inducing microbial aggregation in the centre of the compartment. The experiments so far suggest that the inhibition of photosynthesis leads to a decrease in the velocity of the cells. Nonetheless, under low light conditions, the cells can still perform aerobic respiration to produce energy for self-propulsion and to maintain metabolic functionalities. To examine if and how oxygen respiration affects the aggregation, we developed compartments with different air permeability boundary conditions (electronic supplementary material, figure S1). For compartments with maximum oxygen availability everywhere, no aggregation was observed under low light conditions, while the velocity remained at *v* = (100 ± 10) μm s^−1^ for all cells, indicating that inhibiting only photosynthesis is insufficient to reduce the velocity and induce microbial aggregation. Also in cases of microbial suspensions in compartments without any coupling to an external oxygen reservoir, aggregation was not observed, however the velocity was significantly reduced for all cells to *v* = (63 ± 5) μm s^−1^ (see electronic supplementary material, figure S1). This suggests that under low light condition, cells switch from photosynthesis to respiration, and as the oxygen is depleted the motility of the cells decrease. In summary, the aggregation forms in low light conditions only under inhomogeneous oxygen concentrations, where cells in regions of high oxygen availability (near the lateral boundaries) move faster and remain in a dilute phase, while cells in oxygen-deprived regions (in the centre of the compartment) slow down and aggregate.

**Figure 4.**
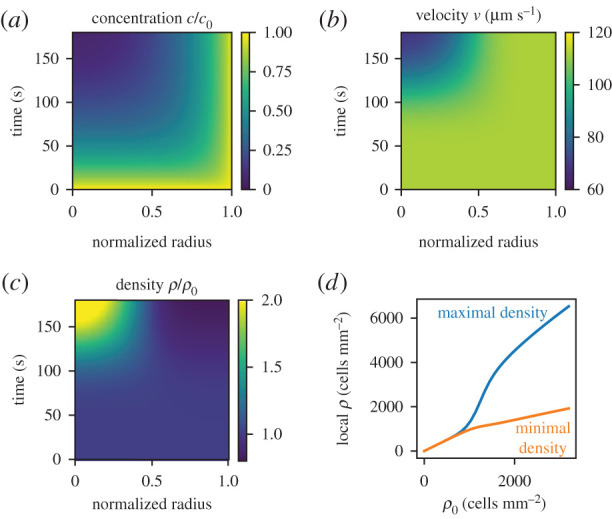
Results of a continuum model for microbial aggregation based on a reaction–diffusion mechanism. (*a*–*c*) Kymographs of the scalar concentration *c*, the cell velocity *v* predicted from equation (7.2), and the cell density *ρ* obeying *ρ* ∝ *v*^−2^. (*d*) Maximal and minimal density in the compartment as a function of the mean density *ρ*.

In order to substantiate these hypotheses, we next introduce a continuum model, which is based on the reaction–diffusion dynamics of the oxygen concentration in the compartment. In particular, we assume that the sidewalls provide an oxygen reservoir, essentially fixing the concentration at the wall. Inside the compartment, cells consume oxygen under low light conditions, leading to an oxygen gradient away from the walls. Our quantitative model combines the observed relation *ρ* ∝ *v*^−2^ of the cell density *ρ* (see figures 1*e* and 2*b*) with an empirically determined velocity–concentration dependence *v*(*c*), which describes the transition from high to low velocities *v* as the local oxygen concentration *c* decreases; see Methods. Starting from homogeneous initial conditions, we then first observe a reduction in the oxygen concentration at the centre of the compartment (figure 4*a*), leading to a local slow–down of the cells (figure 4*b*), which then accumulate (figure 4*c*). Note that the overall consumption of oxygen, and thus its depletion, depends on the mean cell density. In particular, for low cell densities, oxygen is not depleted strongly and all cells have similar (high) velocities yielding a homogeneous distribution. Figure 4*d* shows that the critical density, at which a separation into regions of different densities becomes noticeable, is about 800 cells mm^−2^, similar to the experimental result (figure 2*f*). Taken together, this minimal continuum model demonstrates that the accumulation is driven by the collective oxygen consumption and a local adjustment of the cells’ velocity.

## 6. Discussion

We unravel the interplay of microbial motility and microbial responses to light conditions and local oxygen concentration fields, which enables isolation of the feedback mechanism for the localized aggregation of an ensemble of motile photoactive microbes: under low light conditions, *C. reinhardtii* cells switch their energy production metabolism from photosynthesis to aerobic respiration. If the cell density is sufficiently high and oxygen is not replenished externally at a sufficiently high rate, oxygen is locally deprived resulting in a decrease in microbial swimming velocity. As a result of a generic coupling between cell density and swimming velocity, given through the power law *ρ* ∝ *v*^−2^, microbial aggregation occurs in regions of reduced motility. An inverse density–velocity proportionality has been shown to be able to cause spontaneous aggregation in active matter systems through a positive-feedback mechanism called motility-induced phase separation (MIPS) [33]. However, the strength of the power-law dependence observed in our system is not sufficient for an aggregation to spontaneously form through a MIPS-like phenomenon, but rather appears to be a generic trait of the aggregation mechanism at work. Even though the deprivation of oxygen decreases the swimming velocity of the motile microbes, there is no effect on their tumbling time, which only depends on the cell density. This phenomenon of light-regulated microbial aggregation is a direct consequence of the ability of planktonic photosynthetic microbes to reversibly switch their energy production from photosynthesis to oxygen respiration. Such aggregates of motile microbes form in the complete absence of external light gradients (phototaxis) [11,13,19], nutrient sources (chemotaxis) [34,35], photokinesis [20], aerotaxis (see electronic supplementary material, figure S8) and quorum sensing and thus represent manifestations of another remarkable collective behaviour of motile living cells.

## 7. Methods

### 7.1. Cell cultivation

Wild-type *C. reinhardtii* cells, strain SAG 11-32b (directly provided by the Göttingen Algae Culture Collection, SAG), were grown axenically in tris–acetate–phosphate (TAP) medium on 12 h : 12 h day : night cycles at temperatures of 24°C and 22°C, respectively, in a Memmert IPP110Plus incubator. The daytime light intensity was 28 ± 10 μmol m^−2^ s^−1^, while during the nighttime the light intensity was reduced to zero. Experiments were performed with vegetative cells taken from the cultures in the logarithmic growth phase during the daytime on the third day after incubation. The experimental culture was centrifuged for 10 min at an acceleration of 100*g*, the excess fluid was removed, and the pellet of cells was resuspended in fresh TAP medium. Since cells may deflagellate due to the mechanical shearing during the centrifugation, the cell suspension was allowed to rest for 2 h after centrifugation, which is sufficient to regrow their flagella [36]. In order to control the overall cell concentration in the suspension, a haemocytometer (Neubauer-improved with double net ruling) was used for manual cell counting. The final cell suspension density before experiments ranged between 1 × 10^6^ to 8 × 10^7^ cells ml^−1^.

### 7.2. Experimental set-up

All experiments were performed with an Olympus IX83 inverted microscope and combining LED illumination (CoolLED) with bandpass filters. The condenser was always adjusted to ensure Köhler illumination, which results in about a 5 mm in diameter beam of light on the sample that encompasses the entirety of the compartment. In addition, it provides homogeneous and reproducible light conditions, with the light varying no more than 5% within the compartment. A 4× objective was used in conjunction with the magnification changer set at 1.6× resulting to a total of 6.4× magnification. This configuration provides a large field of view that includes the majority of the compartment (1.95 × 1.95 mm), while it is large enough to perform single-cell tracking. For experiments used to measure the velocity autocorrelation for large densities, a 10× objective was used instead to more accurately identify and track the cells. The compartments for the cell suspensions were made out of polydimethylsiloxane (PDMS). Specifically, the commercially available Sylgard 184 PDMS was used in all experiments, and the mixing ratio used to produce the PDMS is 10 : 1 by weight base to curing agent for all experiments. In order to achieve the desired compartment height, the mixture of PDMS was spin coated on a glass slide at 1300 r.p.m. for 5 min, to achieve the final thickness of (21 ± 1) μm. The glass slide was immediately placed on a hot plate at about 95°C to accelerate the cross-linking process of the PDMS chains. Afterwards, the glass slides were placed in an oven at 75°C for 2 h to complete the polymerization process, and a circular punch of *R* = 1.5 mm (Harris Uni-Core) was used to produce the desired circular compartment. For the experiments, 20 μl of the suspension was pipetted in the compartment and it was sealed with a secondary glass slide. In the case of compartments with high air permeability, a 1 mm thick PDMS cover was used that was prepared by placing liquid PDMS between two glass slides with spacers. The glass slides with the PDMS mixture were placed in the oven at 75°C for 2 h, after which they were peeled off. In the case of compartments with low air permeability, a circular compartment was used made out of stainless steel. A hole of 3 mm in diameter was drilled in a 25 μm thick stainless steel. Since steel does not make a good seal with glass, a homemade clamp was used to ensure a tight fit between the glass slides and the steel compartment.

### 7.3. Light conditions

The light conditions were precisely controlled during all experiments. All experiments regarding the aggregation dynamics and the motility characterization (figures 1 and 3) were performed under red light to avoid adhesion to surfaces [14]. This was achieved using an interference bandpass filter with centre wavelength of 671 nm and a full width at which the intensity is half of the maximum (FWHM) of 10 nm. For the colour discrimination experiments (figure 2), additional interference bandpass filters were used: 523 nm (FWHM: 23 nm), 555 nm (FWHM: 30 nm), 582 nm (FWHM: 10 nm) and 636 nm (FWHM: 20 nm). Wavelengths below 523 nm were not considered since the cells may exhibit both phototaxis [19] and surface adhesion [14]. All light intensities were measured using a Thorlabs PM100D powermeter (Thorlabs S130C photodiode power sensor) for monochromatic light. The light intensities are controlled using a switch implemented in the microscope, which changes the intensity instantly. During the experiment, the light conditions are typically changed every 10 min.

### 7.4. Data recording and cell tracking

All data were recorded using a Photometrics Iris 9 camera at 33 fps. The combination of the relatively high quantum efficiency (greater than 73%) together with the 16-bit depth enabled sufficiently fast data recording at the lowest light intensities. The array size of 2960 × 2960 px enable a large field of view to be imaged to perform the experiments. A background image for each experiment was calculated as the maximum intensity projection of the image sequence. The maximum intensity was selected since cells appear darker in the image. Hence, only stationary cells and objects will appear dark in the background image. The background image was then subtracted from the image sequence to remove detection of any stationary objects. For very low light intensities, there are intensity fluctuations within the image sequence which can diminish particle detection. We solve this issue by scaling the intensity of each image such that the average is constant for each image sequence. Finally, we applied a 2.7 × 2.7 μm median filter.

The cells were detected using the circular Hough transform [37], which only needs a part of the boundary of a circle to detect it. This is important in our case since we lose part of the cell edges when the density becomes sufficiently large. The disadvantage of the circular Hough transform is that it requires a priori knowledge of the expected radius of the objects in the image. In our case, we used a range of radii between 3.3 μm and 7.2 μm. Once the cells have been detected, their trajectories are determined by using a Matlab-based code by Blair & Dufresne [38]. The only parameter needed for the calculation is the maximum distance a particle can travel between frames. We chose this parameter by setting the maximum possible velocity of the cells to be between (133−200) μm s^−1^ depending on the experiment. As a result, the maximum distance a particle can travel between frames is between (4−6) μm, which highly limits the chance of mistracking since the cell diameter is (7−10) μm. If a cell cannot be identified on a particular frame, the track is terminated at that location in order to avoid mistracking that could result on artifacts when calculating the velocity autocorrelation function.

The cell velocity is taken as the displacement of the tracked cells, Δ*x*, over one frame, Δ*t* = 30 ms, and, thus, defined as *v* = Δ*x*/Δ*t*. The local cell density *ρ* is calculated using Voronoi tessellation of the tracked cells in each frame. Voronoi tessellation partitions the space into regions, with each region associated with a single cell. In addition, any point in a given region is closer to the associated cell of that regions compared to any other cell. We define the area of the region associated with *i*th tracked cell as *A*_*i*_, and the corresponding local density as $\rho_{i}=A_{i}^{-1}$. A distribution of all *ρ*_*i*_ is shown in electronic supplementary material, figure S3*d*, while for the data shown in figures 1*e* and 2*a*,*b*, an azimuthal average has been performed.

In order to calculate the average velocity and density as a function of the compartment radius *r* (figures 1*f*,*g*), we average over all particles within an annulus, which has an area of *A* = 2*π* *r* Δ*r*, where Δ*r* is the width of the annulus. In order to have consistent statistics, we keep *A* constant by decreasing Δ*r* with increasing *r* for all annuli, as it can be observed in figures 1*f*,*g*. We use 50 radial bins, which results to Δ*r* varying between 212 μm at the centre of the compartment to 15 μm at the edge.

### 7.5. Chlorophyll absorbance

The absorbance of a substance is defined as *A* = −log_10_ *T*, where *T* = *I*_*T*_/*I*_0_ is the transmittance with *I*_*T*_ and *I*_0_ the transmitted and incident light intensity. We define the absorbed intensity as *I*_*A*_ = *I*_0_ − *I*_*T*_ = *I*_0_(1 − 10^−*A*^). Finally, we use the values of *A* from literature for chlorophyll a and b obtained from *C. reinhardtii* cells [27]. By comparing the detected particles with the original images, we estimated an error in our cell detection. For *ρ* < 1500 cells mm^−2^, we have an error of about 2% in the density, which can become as large as 6% for the highest densities.

### 7.6. Chlorophyll auto-fluorescence

Chlorophyll absorbs mostly at wavelengths around 470 nm and 670 nm, while it emits in the range of (650−750) nm via auto-fluorescence [39]. In the experiment, a 670 nm bandpass filter with a 10 nm FWHM was placed between the light source and the sample, while a 700 nm long-pass filter right after the sample allows only the fluorescent light to be detected by the camera. For all measurements, the camera settings were kept constant. In addition, two experimental measurements were performed for each light intensity: one with the sample and one without, and each measurement consisted of a 300 image sequence at 33 fps. The average intensity projection was calculated for each measurement, and the image acquired without the sample were used to subtract the background noise from the image acquired with the sample. Finally, the average auto-fluorescence signal was then calculated for each light intensity, which is displayed in electronic supplementary material, figure S6.

### 7.7. Continuum model for cell aggregation

Our continuum model for *C. reinhardtii* cell aggregation is based on the hypothesis that the local oxygen concentration regulates the cell motility under low light conditions; a high oxygen concentration implies a high motility, whereas cells slow down in oxygen-depleted regions. Aggregation then occurs in regions with low motility. Empirically, this is supported through the experimentally observed *ρ* ∼ *v*^−2^ density-velocity dependence; see figure 1*e*.

We model the oxygen concentration field *c* in the circular compartment through a two-dimensional reaction–diffusion equation,

7.1 $$\partial_{t}c=D\nabla^{2}c-k\rho c,$$

on a disc of radius *R*. Here, *D* = 2000 μm^2^ s^−1^ is the diffusion constant of oxygen in water [40]; *k* = 10^−5^ mm^2^ s^−1^ denotes the rate of the first-order reaction kinetics, whose value was chosen to match the experimental kymographs. The choice of the reaction kinetics is not very important as we obtain similar results from zeroth-order kinetics. In the experimental set-up, oxygen can diffuse into the compartment through the lateral PDMS walls. For our modelling, this provides the boundary condition *c*(*R*) = *c*_sat_ = 6 × 10^−6^ μmol mm^−2^, basically assuming coupling to a reservoir.

To solve the reaction–diffusion equation, the cell density *ρ* needs to be specified as a function of the local oxygen concentration. As an intermediate step, we infer the velocity–concentration dependence, such that we eventually specify *ρ*(*v*(*c*)). To calibrate our model, we obtain a smoothed density profile *ρ*(*r*) and a velocity profile *v*(*r*) from the experimental measurements and use them as input as explained in the following.

To obtain the steady-state concentration profile *c*(*r*), we solve (7.1) for the measured steady-state density profile *ρ*(*r*). We obtain the velocity–concentration dependence by plotting the model oxygen profile against the measured velocity profile, and find that it can be parameterized by

7.2 $$v(c)=v_{\min}+\frac{v_{\max}-v_{\min}}{2}\left[1+\text{tanh}\left(\frac{c-c_{\mathrm{typ}}}{w}\right)\right].$$

This sigmoidal function interpolates between a minimal velocity *v*_min_ = 56 μm s^−1^ and a maximal velocity *v*_max_ = 112 μm s^−1^, depending on the oxygen concentration difference to a typical concentration *c*_typ_ = 0.14 *c*_sat_. The sharpness of the transition is controlled by the parameter *w*, for which we choose *w* = 0.11 *c*_sat_. As expected, cell motility increases with oxygen concentration. Importantly, this velocity–concentration dependence also enables comparisons between experiments with different cell densities. In particular, the profile explains that no aggregation is expected below a certain mean cell density: at low cell densities the local oxygen concentrations are higher, so the velocities are large everywhere and aggregation is suppressed.

Equations (7.1)–(7.2) together with the experimentally motivated *ρ* ∼ *v*^−2^ density-velocity dependence and the conservation of the mean cell density constitute the full model. We solve the full model as an initial value problem assuming an initially homogeneous cell density and oxygen concentration *c* = *c*_sat_ using a central finite difference scheme [41]. The resulting kymographs are presented in figure 4. Alternatively, we could have directly parameterized *ρ*(*c*) from the steady-state solution of (7.1), which results in similar kymographs. However, this simpler, alternative model would not contain any information about the dependency of the velocity *v* on the oxygen concentration *c*, and therefore would not reflect the main modelling hypothesis that oxygen concentration regulates cell motility, which in turn enables local aggregation.

## Data accessibility

Source data for figures 1–4 will be provided with the paper. In addition, data that support the findings of this study can be accessed from https://doi.org/10.5281/zenodo.4497962. Microscopy images were acquired using ‘Micro-Manager’ (v. 1.4). Background correction microscopy images were obtained using ‘ImageJ (Fiji)’ (v. 2.1.0/1.53c). Experimental data were analysed using ‘MatLab’ (v. 2019a). Cell tracking was performed using a custom MatLab code and also open source MatLab code (‘MatLab particle tracking code repository’, cited in the Methods section). Further data analysis code was custom made. Simulation data were obtained and analysed using Python (v. 3.7) using open source packages (e.g. ‘py-pde’) specified in the Methods section of the paper. All the codes used in the project can be accessed from https://doi.org/10.5281/zenodo.4449791.
